# Neutrophil Heterogeneity Identifies an Association of LAMP1 With Proliferative Lupus Nephritis

**DOI:** 10.1002/eji.70022

**Published:** 2025-08-04

**Authors:** Lennard Ostendorf, Panagiotis Garantziotis, Frank Y. Huang, Georg Schett, Accelerating Medicines Partnership: RA/SLE Network, James A. Lederer, Andrea Fava, Deepak A. Rao, Ricardo Grieshaber‐Bouyer

**Affiliations:** ^1^ Deutsches Rheuma‐Forschungszentrum (DRFZ) an Institute of the Leibniz Association Berlin Germany; ^2^ Department of Nephrology and Medical Intensive Care Charité ‐ Universitätsmedizin Berlin, Corporate Member of Freie Universität Berlin, Humboldt‐Universität zu Berlin, and Berlin Institute of Health Berlin Germany; ^3^ BIH Biomedical Innovation Academy, BIH Charité Junior Clinician Scientist Program Berlin Institute of Health at Charité ‐ Universitätsmedizin Berlin Berlin Germany; ^4^ Division of Rheumatology, Inflammation and Immunity, Brigham and Women's Hospital Harvard Medical School Boston Massachusetts USA; ^5^ Department of Internal Medicine 3 – Rheumatology and Immunology Friedrich‐Alexander‐Universität (FAU) Erlangen‐Nürnberg and Universitätsklinikum Erlangen Erlangen Germany; ^6^ Deutsches Zentrum Immuntherapie (DZI) Friedrich‐Alexander‐Universität (FAU) Erlangen‐Nürnberg and Universitätsklinikum Erlangen Erlangen Germany; ^7^ Division of Rheumatology Department of Internal Medicine V – Hematology Oncology and Rheumatology Heidelberg University Hospital Heidelberg Germany; ^8^ Department of Surgery Brigham and Women's Hospital Boston Massachusetts USA; ^9^ Division of Rheumatology Johns Hopkins University School of Medicine Baltimore Maryland USA

## Abstract

Lupus nephritis (LN) is a severe manifestation of systemic lupus erythematosus (SLE) with limited biomarkers for early detection. While neutrophils contribute to SLE pathogenesis, their phenotypic heterogeneity in disease remains poorly characterized. Here, we used mass cytometry to profile blood neutrophils from patients with biopsy‐confirmed proliferative LN and healthy controls. We identified a distinct population of activated neutrophils, marked by surface expression of lysosomal‐associated membrane protein 1 (LAMP1/CD107a), that was virtually absent in healthy individuals. We demonstrate that LAMP1 resides intracellularly in resting neutrophils and translocates to the cell surface upon activation. Transcriptomic analysis revealed no difference in LAMP1 mRNA expression between patients with SLE and controls, confirming that surface LAMP1 reflects neutrophil activation rather than increased transcription. Soluble LAMP1 was significantly elevated in serum from patients with SLE compared with controls, with the highest levels in proliferative LN. In a large cohort of 225 patients with LN, urinary LAMP1 correlated with glomerular filtration rate, proteinuria, and histological activity indices. Together, our findings reveal LAMP1 as a marker of neutrophil activation in SLE and identify serum and urinary LAMP1 as potential noninvasive biomarkers for proliferative LN.

## Graphical abstract

We investigated neutrophil heterogeneity in patients with systemic lupus erythematosus and identified increased surface levels of LAMP1 (CD107a). Stimulation of healthy donor neutrophils led to increased surface LAMP1. Urine and serum soluble LAMP1 levels were increased in patients, especially those with proliferative lupus nephritis.

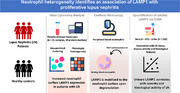

## 1 Introduction

Systemic lupus erythematosus (SLE) is an autoimmune disease characterized by the generation of adaptive immune responses against nuclear antigens [1, 2]. The increased availability of nuclear antigens emerging from increased cell death and impaired clearance leads to excessive formation of type I interferons [3] via activation of nucleic acid‐sensing innate receptors in plasmacytoid dendritic cells and other myeloid cells and, in consequence, to a breach in immune tolerance with formation of autoantibodies. Clinically, SLE can affect almost any organ—often involving the skin, joints, serous membranes, and kidneys. However, clinical manifestations grossly differ between individual patients, and predicting future severe organ manifestations has proven difficult so far. Lupus nephritis (LN) is a type of glomerulonephritis caused by SLE and affects approximately half of patients. LN puts patients at risk for kidney failure, is associated with excess mortality, and usually requires more intensive immunosuppressive treatment [4].

Lupus nephritis is typically classified via histology, with focal glomerulonephritis (involving up to 50% of total glomeruli; class III) and diffuse glomerulonephritis (involving more than 50% of total glomeruli; class IV) being referred to as “proliferative” glomerulonephritis. While some urine‐based biomarkers for lupus nephritis, such as urinary T cells, CD163, CD206, or IL‐16 have been defined [5, 6, 7], the diagnosis of lupus nephritis still relies on an invasive kidney biopsy, and none of the investigated biomarkers have been translated into clinical routine.

Similar to the clinical heterogeneity, immunological investigations have identified a large variance in the immunopathology of SLE: Features like the increased presence of plasmablasts, the type I interferon signature, or increased granulopoiesis can be dominant in some patients but are virtually absent in others. While many studies have attempted to define immunological “endotypes” of SLE that correlate with specific clinical manifestations and disease severity, they have so far not been translated into clinical practice [8, 9, 10, 11].

Neutrophils are strongly implied in the pathogenesis of SLE [12]. Neutrophil‐derived neutrophil extracellular traps (NETs) are an important source of nuclear antigens that perpetuate the adaptive immune response against nucleic acids. (13, 14) Low‐density neutrophils (LDNs), that is, neutrophils that separate with mononuclear cells after density gradient separation, have been identified as an immunological feature of SLE [15] and other immune‐mediated diseases [16, 17, 18]. In SLE, LDN has been described to undergo spontaneous NETosis and produce high levels of proinflammatory cytokines, including type I interferon and TNF, thereby contributing to SLE pathogenesis [15]. Given their contributions to the pathophysiology, neutrophil states could therefore serve as important markers of disease activity or endotypes in SLE. However, due to their short lifespan and limited dichotomous surface markers, in‐depth analysis of neutrophil phenotypes has been challenging [15, 19]. Recent studies have shown how neutrophil phenotypes vary depending on their age, maturity, activation state, and local environment [20, 21].

Here, we performed a mass cytometry analysis of blood leukocytes as part of the AMP RA/SLE consortium in order to explore neutrophil heterogeneity in SLE and identify potential biomarkers for molecular patient stratification. Combining these data with transcriptomic analyses, flow cytometry, confocal microscopy, urine proteomics, and serum ELISA, we identified Lysosomal‐associated membrane protein 1 (LAMP1) as a differentially abundant protein on the surface of neutrophils from lupus patients and identified soluble LAMP1 in urine and serum as correlating with proliferative lupus nephritis.

## 2 Methods

### 2.1 Research Subjects

Patients and healthy subjects were recruited through the Accelerating Medicines (AMP) RA/SLE Partnership Consortium (10 sites in the United States) [22]. Additional patients and healthy donors were recruited at Heidelberg University Hospital and at Erlangen University Hospital. The study was approved by the respective local ethics committes. Patient characteristics and clinical assessments, including measurement of clinical scores and routine serology, were performed at each site. All patients with SLE met the 1997 ACR classification criteria for SLE [23]. LN diagnosis was confirmed by kidney biopsy and graded according to the 2003 ISN/RPS LN classification system [24]. Disease activity was assessed using the Systemic Lupus Erythematosus Disease Activity Index 2000 (SLEDAI‐2K) [25]. Patient characteristics are displayed in Tables S1–S3.

### 2.2 Cytometry by Time of Flight (CyTOF)

Mass cytometry (CyTOF) was performed on cryopreserved whole leukocytes from patients and healthy donors (HD) enrolled in the AMP RA/SLE Network as described previously [20]. Briefly, blood was collected into lithium heparin tubes and erythrocytes lysed using Ammonium‐Chloride‐Potassium Lysing Buffer. Leukocytes were washed in PBS and cryopreserved in a 10% DMSO–containing solution for batched analyses. Cryopreserved whole leukocytes were later thawed into RPMI Medium 1640 (Invitrogen, catalog 11875‐085) supplemented with 5% heat‐inactivated FBS (Invitrogen, catalog 16000044), 1 mM GlutaMAX (Invitrogen, catalog 35050079), antibiotic‐antimycotic (Invitrogen, catalog 15240062), 2 mM MEM nonessential amino acids (Invitrogen, catalog 11140050), 10 mM HEPES (Invitrogen, catalog 15630080), 2.5 × 10–5 M 2‐mercaptoethanol (MilliporeSigma, catalog M3148), 20 units/mL sodium heparin (MilliporeSigma, catalog H3393), and 25 units/mL benzonase nuclease (MilliporeSigma, catalog E1014). Cells were counted, and 0.5 × 10^6^ to 1 × 10^6^ cells from each sample were transferred to a polypropylene plate for staining. The samples were spun down and aspirated. A total of 5 µM of cisplatin viability staining reagent (Fluidigm, catalog 201064) was added for 2 min and then diluted with culture media. After centrifugation, Human TruStain FcX Fc receptor blocking reagent (BioLegend, catalog 422302) was used at a 1:100 dilution in CSB (PBS with 2.5 g BSA [MilliporeSigma, catalog A3059] and 100 mg of sodium azide [MilliporeSigma, catalog 71289]) for 10 min, followed by incubation with conjugated surface antibodies (Table S4) for 30 min. All antibodies were obtained from the Harvard Medical Area CyTOF Antibody Resource and Core.

A total of 16% stock paraformaldehyde (Thermo Fisher Scientific, catalog O4042‐500) dissolved in PBS was used at a final concentration of 4% formaldehyde for 10 min to fix the samples before permeabilization with the FoxP3/Transcription Factor Staining Buffer Set (Thermo Fisher Scientific, catalog 00‐5523‐00). The samples were incubated with SCN‐EDTA coupled palladium–based barcoding reagents for 15 min and then combined into a single sample. Conjugated intracellular antibodies were added to each tube and incubated for 30 min. Cells were then fixed with 1.6% formaldehyde for 10 min.

DNA was labeled for 20 min with an 18.75 µM iridium intercalator solution (Fluidigm, catalog 201192B). Samples were subsequently washed and reconstituted in Milli‐Q filtered distilled water in the presence of EQ Four Element Calibration beads (Fluidigm, catalog 201078) at a final concentration of 1 × 10^6^ cells/mL. Samples were acquired on a Helios CyTOF Mass Cytometer (Fluidigm). The raw FCS files were normalized to reduce signal deviation between samples over the course of multiday batch acquisitions, utilizing the bead standard normalization method established by Finck et al. [26]. These normalized files were then deconvoluted into individual sample files using a single‐cell–based debarcoding algorithm established by Zunder et al. [27]. In the validation cohort, the normalized files were also compensated with a panel‐specific spillover matrix to subtract cross‐contaminating signals, utilizing the CyTOF‐based compensation method established by Chevrier et al. [28].

### 2.3 Analysis of CyTOF Data

Mass cytometry data were gated to exclude debris and identify DNA+ events. Nonviable cisplatin^+^ cells and equalization beads were excluded. FCS files were imported in R using the flowCore package (version 2.16.0) [29], downsampled to 20,000 cells per sample, and combined into a single SingleCellExperiment object. FlowSOM and ConsensusClusterPlus were performed using the CATALYST package (version 1.28.0 [30]). Using the UMAP (Uniform Manifold Approximation and Projection) dimensionality reduction, we identified neutrophils based on the expression of canonical markers and absence of lineage markers for other cell types. (Figure S1A,B) After clustering and dimensionality reduction of the events corresponding to neutrophils, we analyzed the median expression of markers and differential abundance of the *k* = 10 metaclusters. (Figure S1C) Statistical analyses and graphical summaries were produced with the ggpubr package in R version 4.3.2 as appropriate [31].

### 2.4 Transcriptional Analysis of *LAMP1*

We accessed transcriptional data from a previously published SLE cohort dataset of 58 healthy controls and 142 patients with SLE, published in the European Genome‐Phenome Archive (EGA), under the accession number EGAS00001003662 [32]. Normalized *LAMP1* Gene Expression (Counts per million) was compared between groups with the nonparametric Mann–Whitney U test.

### 2.5 Flow Cytometry and Stimulation for the Analysis of LAMP1 Subcellular Location

Blood neutrophils of healthy controls were isolated using density gradient centrifugation as described [21] and stained using anti‐CD107a (LAMP1) AF488 (Clone H4A3, Biolegend #328610), anti‐CD11b AF594 (Clone ICRF44, Biolegend # 301340), and anti‐CD15 BV421 (Clone W6D3, Biolegend #323040) antibodies. For intracellular staining, cells were permeabilized using a 0.3 % saponin buffer and stained with the same antibodies. After staining, the cells were fixed in 2% Paraformaldehyde (PFA) for 30 min and washed with phosphate‐buffered saline (PBS). For the stimulation experiments, isolated neutrophils were suspended in RPMI 1640 Medium (ThermoFischer) and plated at 2 × 10^6^ cells/well in a 96‐well plate. The cells were stimulated with N‐Formylmethionine‐leucyl‐phenylalanine (fMLP, SigmaAldrich #F3506) at 0.1 or 1 µM concentrations and in the presence or absence of Cytochalasin B (SigmaAldrich #C8273) at 37°C for 15 or 30 min as indicated. Directly after stimulation, the cells were fixed in 4% PFA for 20 min. After washing, the cells were stained using CD66b PerCP‐Cy5.5 (Clone G10F5, Biolegend #305108), CD15 AF647 (Clone W6D3, Biolegend #323012), LAMP1 AF488 (Clone H4A3, Biolegend #328610), and CD11b AF594 (Clone ICRF44, Biolegend # 301340) antibodies. The cells were washed and then acquired using a BD LSR II cytometer (Becton, Dickinson and Company), and the data were analyzed using FlowJo (version 10.08.2 for MacOS, FlowJo LLC).

### 2.6 Confocal Microscopy

Neutrophils were plated on glass slides for 15 min. All following steps were performed at room temperature. Confocal immunofluorescence microscopy was performed as previously described: PMNs from healthy donors were isolated as described above and plated for 15 min. Cells were washed with PBS and fixed in 1.5% PFA (paraformaldehyde) overnight. The cells were then permeabilized with 0.1% Saponin buffer and stained with antibodies against LAMP1 (AF488), CD15 (AF594), CD35 (AF647), lactoferrin (LTF; AF647), myeloperoxidase (MPO; AF647), and DAPI. Images were acquired using a Nikon A1R plus N‐SIM confocal laser scanning microscope using a 20× objective, as previously described [33].

Image overlays were produced using fiji (based on ImageJ2 version 4.12.0) [34]. The colocalization analysis of LAMP1 and granule markers was performed using scikit‐image (version 0.25.2) [35] and NumPy (version 2.2.0) [36]. Background subtraction was applied using a rolling ball algorithm (radius 50 pixels). Using the Mesmer algorithm (37), binary masks were produced of the cells. The signal intensity was normalized between 0 and 1. Pearson's correlation coefficient was calculated for 3 fields of view for each costaining to assess the overall signal correlation between the markers.

### 2.7 Quantification of Soluble LAMP1 via ELISA

Quantification of LAMP1 in serum samples was performed using the human lysosomal‐associated membrane protein 1 (LAMP1) ELISA Kit (Mybiosource cat # MBS2023492). The detection range for the ELISA Kit is reported as 0.156–10 ng/mL, and the sensitivity is <0.061 ng/mL.

### 2.8 Urine Proteomics

Urine proteomic data were generated using the Kiloplex Quantibody (RayBiotech) as previously described [7, 38]. Urine protein abundances were calculated and expressed as pg_protein_/mg_creatinine_.

## 3 Results

### 3.1 Neutrophil Surface LAMP1 Is Increased in Patients With SLE and Lupus Nephritis

**FIGURE 1:**
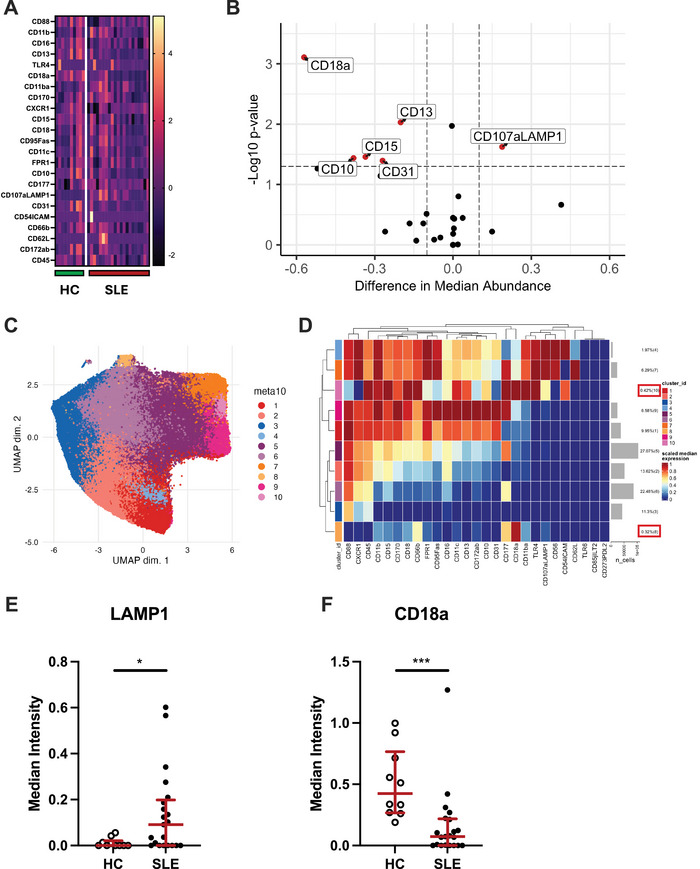
Mass cytometry analysis of blood leukocytes identifies increased expression of LAMP1 in patients with lupus nephritis compared with healthy donors. (A) Heatmap of the expression of 23 surface proteins on blood neutrophils from healthy donors (*n* = 10) and patients with LN (*n* = 21). (B) Volcano plot showing differential abundance of the examined surface markers in blood leukocytes in LN patients versus healthy donors. Positive difference in median abundance values indicates higher abundance in LN patients. (C) UMAP dimensionality reduction of the mass cytometry data of blood neutrophils from LN patients and healthy donors in 10 neutrophil meta‐clusters. (D) Marker protein expression and relative cell abundance of the neutrophil meta‐clusters. Clusters #8 and #10, which are increased in LN patients, are shown in the red boxes. (E, F) Abundance of LAMP1 (CD107a) (E) and CD18a (F) on neutrophils of patients with LN and healthy donors. Mann–Whitney *U* test: *p < 0.05; ***p < 0.001. UMAP: Uniform Manifold Approximation and Projection.

We investigated the phenotypic heterogeneity of peripheral blood neutrophils from patients with lupus nephritis (LN) (*n* = 21) and healthy donors (*n* = 10) [22]. A number of neutrophil surface markers, including CD10, CD16, and CD18a, which are associated with neutrophil aging, maturation, and activation status [39], showed gradual variation in signal intensity and demonstrated the heterogeneity of neutrophils in SLE (Figure S1C). Analyzing median signal intensities, we found 6 of 34 analyzed markers to be significantly different between lupus nephritis and HD (Figure 1A,B).

To further investigate neutrophil diversity in SLE, we performed unsupervised FlowSOM and ConsensusClusterPlus metaclustering, identifying 10 meta‐clusters (Figure 1C,D). Comparing their relative abundance in patients with lupus nephritis and healthy subjects, two clusters were significantly more abundant in patients with lupus nephritis (Clusters #8 and #10). Interestingly, both were low‐abundance clusters that were virtually absent in healthy subjects and were marked by high levels of neutrophil activation markers (e.g., CD54, CD18a), indicating that circulating activated neutrophils can be identified in LN patients.

On an individual marker level, the largest differences between healthy subjects and patients with lupus nephritis were a decrease in CD18a (median signal intensity 0.165 (LN) versus 0.859 (HD)) and an increase in LAMP1 in patients with lupus nephritis compared with healthy subjects (median signal intensity 0.170 (lupus nephritis) versus 0 (healthy subjects)) (Figures 1E,F **)**.

### 3.2 LAMP1 is Expressed Intracellularly in Resting Neutrophils and Mobilized to the Surface Upon Degranulation

**FIGURE 2:**
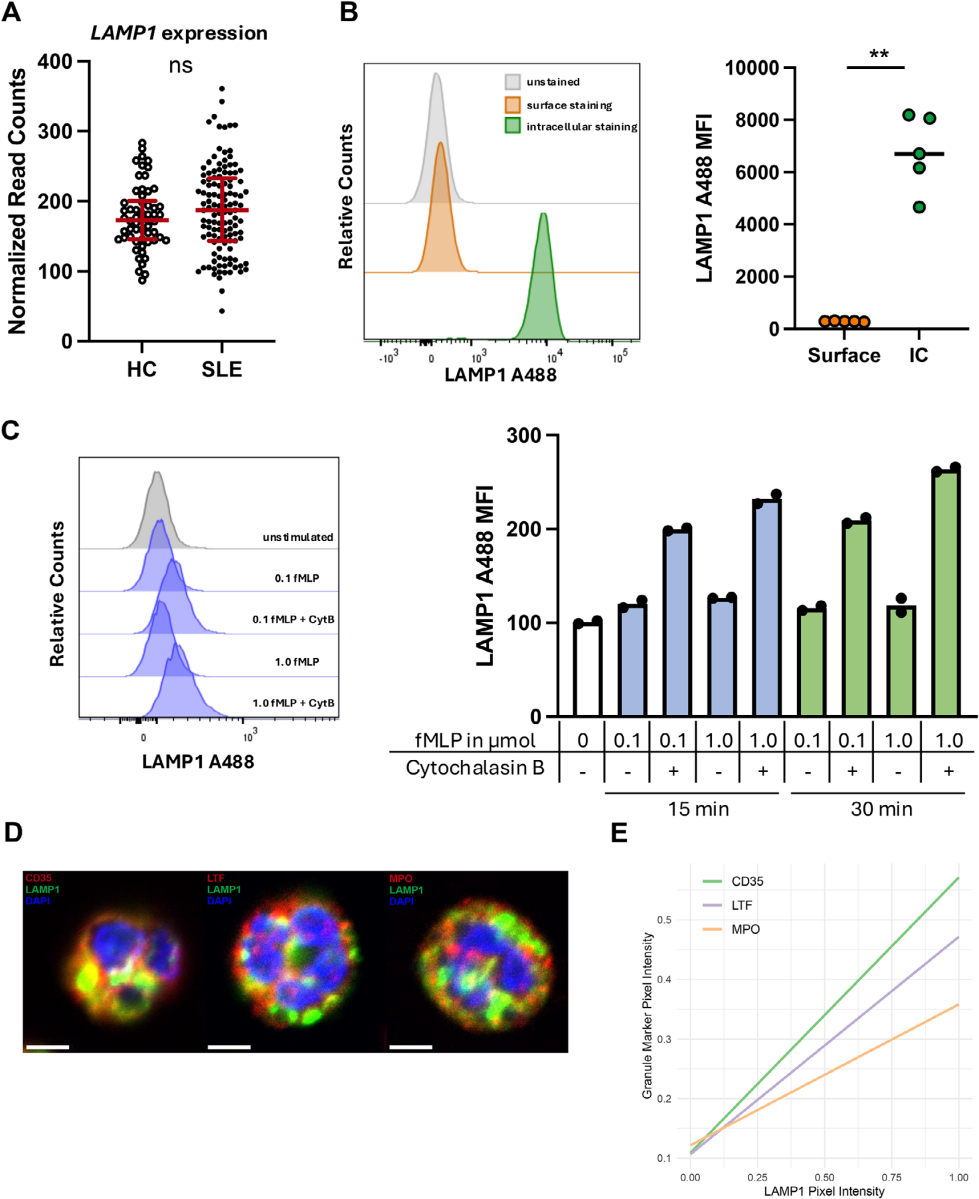
LAMP1 is stored intracellularly in secretory vesicles and mobilized upon neutrophil activation. (A) *LAMP1* mRNA expression in peripheral blood from 58 healthy donors and 142 patients with SLE. (B) Surface and intracellular flow cytometry of LAMP1 in neutrophils from a healthy donor. Quantification of LAMP1 MFI on the neutrophil surface before and after permeabilization (healthy blood donors, *n* = 5). (C) Surface LAMP1 MFI of unpermeabilized neutrophils from healthy blood donors after incubation with fMLP and Cytochalasin B. (D) Confocal microscopy of healthy donor neutrophils, costained for LAMP1 and granule markers MPO (primary/azurophilic granules), LTF (secondary/specific granules), and CD35 (secretory granules). Scale bar: 5 µm. (E) Colocalisation of LAMP1 with granule markers calculated as Pearson correlation. Mann–Whitney *U* test: **p < 0.01. MFI, Median fluorescence intensity.

We then investigated the mechanism of increased LAMP1 signal on the neutrophil surface in LN patients. LAMP1 is typically found within intracellular organelles such as the lysosome [40]. We first questioned whether the increased LAMP1 detection reflected increased transcription or translocation of LAMP1 to the cell surface. Therefore, we analyzed *LAMP1* gene expression in a blood transcriptome dataset of 58 healthy controls and 142 patients with SLE [32]. No significant difference in *LAMP1* expression was identified between the groups (Figure 2A). Specifically, *LAMP1* expression was not different between patients with and without LN or between active and inactive lupus nephritis (Figure S2).

We thus hypothesized that the increased signal intensity of LAMP1 could result from its translocation to the cell surface, as has been described for lymphocytes [41]. To this end, we confirmed the intracellular localization of LAMP1 in blood neutrophils from healthy donors by performing both surface and intracellular staining. In resting neutrophils, LAMP1 was localized predominantly intracellularly, with minimal surface expression (Figure 2B). To test if neutrophil activation could mobilize LAMP1, we stimulated blood neutrophils with fMLP and Cytochalasin B. The combination of fMLP and Cytochalasin B resulted in a significant increase in surface LAMP1 expression (Figure 2C), suggesting that activation‐mediated externalization explains the observed increase in LAMP1 on LN neutrophils.

We next applied confocal microscopy to examine the intracellular distribution of LAMP1 in neutrophils from healthy donors (Figure 2D **;** Figure S3). We assessed the colocalization of different granule markers (myeloperoxidase [MPO] for primary/azurophilic granules, lactoferrin [LTF] for secondary/specific granules, CD35 for secretory granules). Only partial colocalization of LAMP1 was observed with all granule markers, consistent with the reported lysosomal localization of LAMP1 [42]. Colocalization analysis of the granule markers and LAMP1 within cells revealed the strongest colocalization of LAMP1 with CD35 (Pearson correlation r 0.461), suggesting the presence of LAMP1 in secretory vesicles. The colocalization with lactoferrin (0.393) and MPO (0.328) was lower, respectively (Figure 2E).

Taken together, these findings suggest that the increased abundance of LAMP1 on LN neutrophils is not caused by increased transcription, but by neutrophil activation and translocation of LAMP‐1 to the cell surface.

### 3.3 Soluble LAMP1 Is Increased in the Serum of Patients With SLE

**FIGURE 3:**
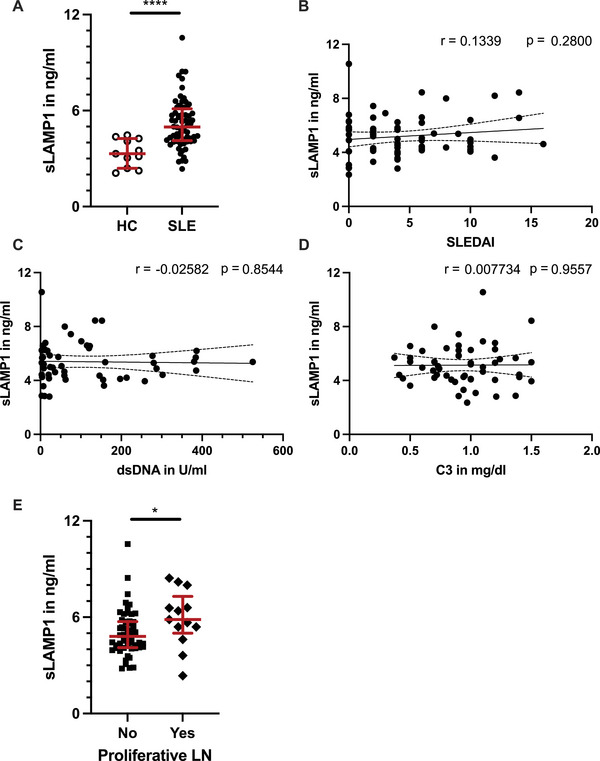
Soluble LAMP1 is increased in the serum of patients with SLE. (A) Soluble LAMP1 (sLAMP1) measured by ELISA in the serum of healthy donors (*n* = 11) and patients with SLE (*n* = 67). (B–D) Pearson correlation of serum LAMP1 with SLEDAI‐2K. (B) dsDNA autoantibodies (C), serum C3. (D) levels in patients with SLE. (E) Soluble LAMP1 is higher in patients with proliferative (class III/IV) LN compared with patients without proliferative LN. Mann–Whitney *U* test: *p < 0.05; ****p < 0.0001.

Since LAMP1 is externalized upon neutrophil activation, we hypothesized that LAMP1 might also be released in soluble form into the circulation. We quantified soluble LAMP1 in the sera of 67 patients with SLE and 11 healthy subjects using ELISA (patient characteristics in Table S2). Similar to neutrophil surface LAMP1 expression, serum concentrations of soluble LAMP1 were markedly increased in patients with SLE (median soluble LAMP1: 4.98 ng/mL) compared with healthy subjects (3.30 ng/mL; Figure 3A). While the median age of healthy donors in the available serum cohort was higher than that of the patients with SLE, we found no significant correlation between age and serum LAMP1 (Figure S3A). In addition, there was no significant correlation of LAMP1 with prednisolone dose or other treatments (Figure S3B).

We then tested if serum LAMP1 levels correlated with clinical characteristics in patients with SLE. While serum LAMP1 levels were elevated in SLE in general, they did not correlate with current disease activity as measured by the SLE disease activity index (SLEDAI‐2K), nor did they correlate with serological markers of SLE activity, such as dsDNA antibody levels or complement levels (Figures 3B–D). However, we identified increased serum LAMP1 levels in patients with proliferative (i.e., class III or IV) LN (Figure 3E) compared with patients with SLE without proliferative LN, suggesting it might be able to identify and/or monitor renal involvement.

### 3.4 Urinary LAMP1 Correlates With Kidney Function and Nephritis Activity

**FIGURE 4:**
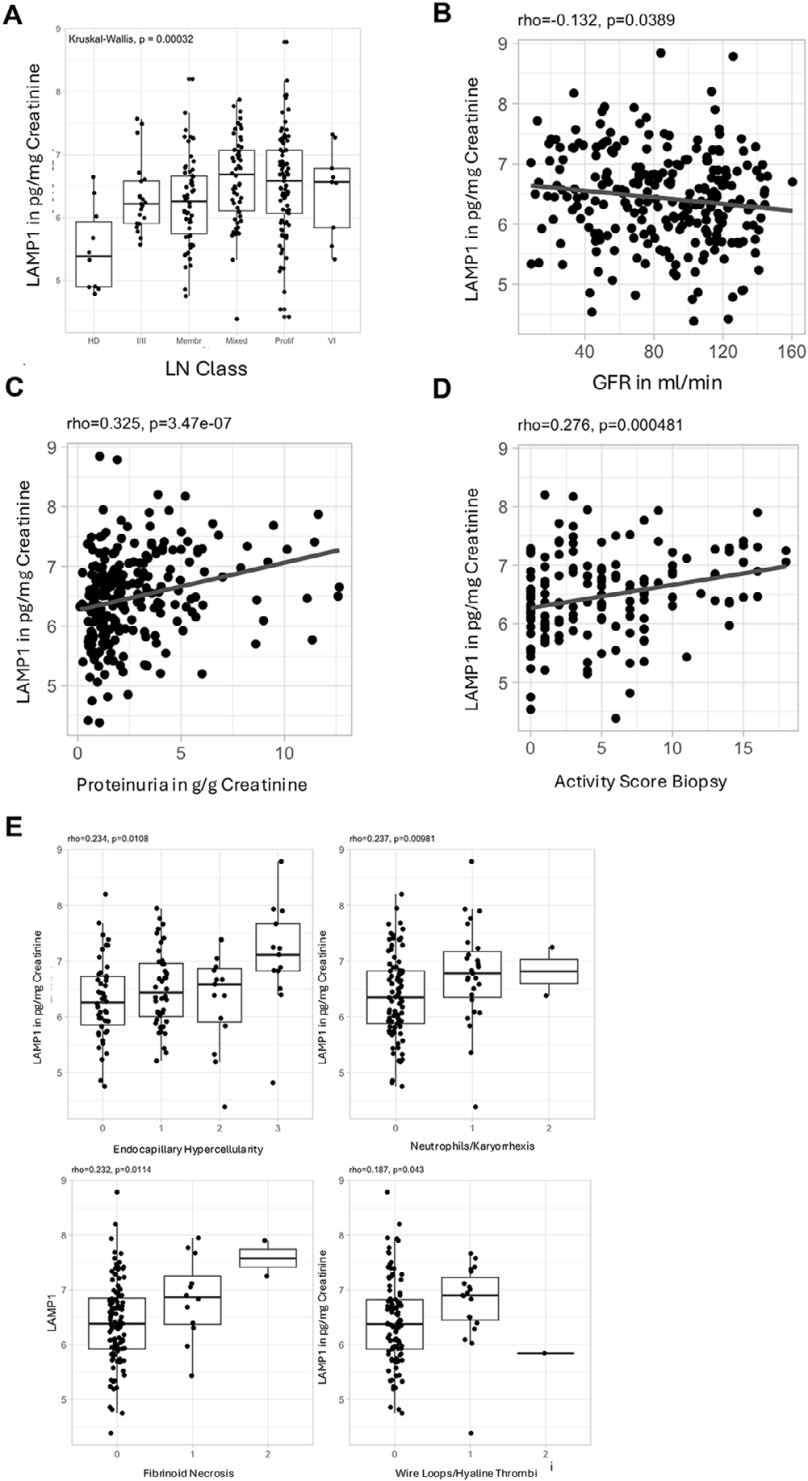
Urinary LAMP1 correlates with nephritis severity and histological activity in LN. (A) Urinary LAMP1 levels in healthy donors (*n* = 10) and LN patients (*n* = 225). (B–D) Correlation of (B) glomerular filtration rate (GFR), (C) proteinuria (g/g creatinine), and (D) Biopsy NIH activity index (D) with urinary LAMP1 (in pg/mg creatinine). (E) Correlation of LAMP1 with specific features of the NIH activity index of kidney biopsies.

Given that serum soluble LAMP1 was elevated in patients with proliferative LN and considering that LN is characterized by increased myeloid cell activation in the kidney, we hypothesized that LAMP1 might also be released into the urine. To test this, we analyzed soluble LAMP1 levels in urine proteomic data from 225 LN patients [38]. Soluble LAMP1 was detected in the urine and was significantly increased in patients with LN compared with healthy donors (Figure 4A).

Amongst LN patients, urinary LAMP1 levels were higher in patients with proliferative LN (class III/IV) compared with those with I/II or pure membranous (class V) LN (Figure 4A). Urinary LAMP1 showed a significant correlation with both glomerular filtration rate and proteinuria (Figures 4B–C). Consistent with these findings, urinary LAMP1 also correlated with the LN biopsy NIH activity index [43] (Figure 4D), specifically the activity index components endocapillary hypercellularity, neutrophil infiltration/karyorrhexis, fibrinoid necrosis, and hyaline thrombi/wire loops in the kidney biopsies (Figure 4E). These findings indicate a potential direct link between glomerular leukocytes and urinary LAMP1 levels, suggesting it could serve as a noninvasive biomarker of kidney involvement in SLE.

## 4 Discussion

In a comprehensive analysis of neutrophil phenotypes, we identified LAMP1 (CD107a) as a differentially expressed neutrophil marker in patients with lupus nephritis. Mechanistic experiments confirmed that under physiological conditions, LAMP1 is found in intracellular organelles, in particular in secretory vesicles, while neutrophil stimulation increases surface expression of LAMP1. In addition, soluble LAMP1 was increased in the serum and urine of patients with lupus nephritis and correlated with proteinuria and inflammatory activity in the kidney biopsy.

LAMP1 belongs to the family of lysosome‐associated membrane glycoproteins, which are strongly glycosylated proteins crucial for preserving lysosomal pH and membrane integrity [44]. “Lysosomal fragility” has been hypothesized to be relevant to the pathogenesis of SLE [45]. Lysosomes are actively engaged in the recognition of pathogens, activation of pattern recognition receptors, processing of antigens for presentation on MHC molecules, clearance of apoptotic debris, and regulation of cytokine release, processes relevant in SLE. Dysregulation of lysosomal enzymes has been reported in SLE [46]. LAMP1 can also be found on the cellular surface when lysosomes fuse with the cell membrane, promoting adhesion of PBMCs to the vascular endothelium. [41, 44]. Additionally, LAMP1 is often used as a degranulation marker for cytotoxic lymphocytes [47], while it has been less investigated in myeloid cells.

Here, we identified increased LAMP1 surface expression after neutrophil activation. This increase in surface LAMP1 could be caused by multiple mechanisms. One previous study reported LAMP1 on the surface of pro‐inflammatory, low‐density granulocytes (LDGs) in patients with SLE [48]. These LDGs were found to have increased expression of CD63 (LAMP3), another lysosomal glycoprotein, which is an established activation marker of granulocytes [48]. Another potential mechanism is linked to neutrophil aging. Circadian neutrophil aging has also been associated with gradual degranulation, leading to a less inflammatory potential [49]. In our data, neutrophils from patients with SLE had an overall less mature phenotype, with lower expression of markers associated with neutrophil maturity, such as CD10 and CD13.

We also showed an increase of soluble LAMP1 in SLE serum and urine. Systemically increased LAMP1 indicates a generalized increase in neutrophil activation in patients with lupus nephritis, likely reflecting processes such as NETosis, which might contribute to LAMP1 release. In a proteomic analysis of the urine samples of a large cohort of patients with lupus nephritis, LAMP1 was associated with the severity of inflammatory activity in the kidney biopsy. Only 127 (including LAMP1) of 1200 urinary proteins were significantly correlated with the NIH Activity Index, indicating that LAMP1 is specifically associated with LN activity [38]. Notably, total proteinuria was not associated with histological activity. In previous work, we showed that the decline of urinary biomarkers of histological activity in responders was not dependent on the general decline of proteinuria since the concentration of many average control proteins remained flat in responders and nonresponders [50]. Association with biopsy features such as endocapillary proliferation and glomerular neutrophils/karyorrhexis additionally points to the glomerular activation and degranulation of neutrophils and other immune cells as a source of urinary LAMP1. Thus, while LAMP1 itself likely does not directly influence lupus pathophysiology, it indicates the increased activation and degranulation of neutrophils and potentially other immune cells, including in inflamed tissues. Future investigations should elucidate the stimuli leading to degranulation and LAMP1 externalization in SLE.

The routine diagnosis and grading of lupus nephritis are still dependent on invasive kidney biopsy. Proteinuria, while used for longitudinal assessment of lupus nephritis severity and treatment response, has been shown to poorly differentiate disease activity from persistent damage [38] and to poorly reflect LN biopsy classes [51, 52]. Hence, biomarkers that can be applied noninvasively and longitudinally are urgently needed. Soluble LAMP1 was increased in the urine and serum of patients with lupus nephritis, and was especially high in patients with proliferative (class III/IV) LN. Its potential to identify kidney involvement, especially in concert with other biomarkers, should be further investigated. Our study has a number of limitations: While we identified increased LAMP1 on neutrophils and increased soluble LAMP1, we cannot definitely attribute the source of the soluble LAMP1 to neutrophils alone; other degranulating or disintegrating cells could also contribute to the measured increase. In addition, we cannot rule out the influence of immunosuppressive treatment on LAMP1 levels. The median age of healthy donors was higher than that of the patients with SLE in the serum cohort; however, we did not find a significant correlation between age and serum LAMP1. Lastly, since the urinary LAMP1 cohort did not include patients with SLE without LN, it remains unclear whether these findings are specific to LN compared with other inflammatory kidney diseases and compared with SLE without kidney involvement.

In summary, our study revealed an increase in neutrophilic surface and soluble LAMP1 in SLE, especially in patients with proliferative LN, with potential as a noninvasive biomarker of kidney disease.

## Author Contributions

Ricardo Grieshaber‐Bouyer, Frank Y. Huang, and Andrea Fava performed experiments. Lennard Ostendorf, Panagiotis Garantziotis, Andrea Fava, and Ricardo Grieshaber‐Bouyer analyzed the data. Georg Schett, James A. Lederer, and the Accelerating Medicines Partnership Network provided clinical samples and generated experimental data. Deepak A. Rao and Ricardo Grieshaber‐Bouyer supervised the work. Lennard Ostendorf, Panagiotis Garantziotis, and Ricardo Grieshaber‐Bouyer wrote the first draft of the manuscript. All authors gave critical input toward the manuscript and approved the final version.

## Conflicts of Interest

The authors declare no conflicts of interest.

## Peer Review

The peer review history for this article is available at https://publons.com/publon/10.1002/eji.70022.

## Supporting information




**Supporting File 1**: eji70022‐sup‐0001‐SuppMat.pdf

## Data Availability

CyTOF data are accessible via ImmPort, Study number 997: https://www.immport.org/shared/study/SDY997. Proteomics Kiloplex data is available at synapse.org under the accession code: syn61515624.
