# Supplementary material for: Neutrophil Heterogeneity Identifies an Association of LAMP1 With Proliferative Lupus Nephritis
Source: Eur J Immunol. 2025 Aug 4;55(8):e70022. doi: 10.1002/eji.70022 (PMC12322514; doi:10.1002/eji.70022)

Supplementary Information for
Neutrophil heterogeneity identifies an association of LAMP1 with proliferative lupus nephritis

Includes:

Members of the Accelerating Medicines Partnership: RA/SLE Network

Supplementary Table 1-4

Supplementary Figure 1-4

Members of the Accelerating Medicines Partnership: RA/SLE Network

Jennifer Albrecht, Jennifer H. Anolik, William Apruzzese, Jennifer L. Barnas, Joan M. Bathon, Ami Ben-Artzi, Brendan F. Boyce, David L. Boyle, Michael B. Brenner, S. Louis Bridges Jr., Vivian P. Bykerk, Debbie Campbell, Arnold Ceponis, Adam Chicoine, Michelle Curtis, Kevin D. Deane, Edward DiCarlo, Laura T. Donlin, Patrick Dunn, Andrew Filer, Hayley Carr, Gary S. Firestein, Lindsy Forbess, Laura Geraldino-Pardilla, Susan M. Goodman, Ellen M. Gravallese, Peter K. Gregersen, Joel M. Guthridge, Maria Gutierrez-Arcelus, V. Michael Holers, Diane Horowitz, Laura B. Hughes, Lionel B. Ivashkiv, Kazuyoshi Ishigaki, Judith A. James, A. Helena Jonsson, Joyce B. Kang, Gregory Keras, Ilya Korsunsky, Amit Lakhanpal, James A. Lederer, Miles J. Lewis, Yuhong Li, Katherine Liao, Arthur M. Mandelin II, Ian Mantel, Kathryne E. Marks, Mark Maybury, Andrew McDavid, Mandy J. McGeachy, Joseph R. Mears, Nida Meednu, Nghia Millard, Larry Moreland, Saba Nayar, Alessandra Nerviani, Dana E. Orange, Harris Perlman, Costantino Pitzalis, Javier Rangel-Moreno, Soumya Raychaudhuri, Karim Raza, Yakir Reshef, Christopher Ritchlin, Felice Rivellese, William H. Robinson, Laurie Rumker, Ilfita Sahbudin, Saori Sakaue, Jennifer A. Seifert, Dagmar Scheel-Toellner, Anvita Singaraju, Kamil Slowikowski, Melanie Smith, Darren Tabechian, Paul J. Utz, Gerald F. M. Watts, Kevin Wei, Kathryn Weinand, Dana Weisenfeld, Michael Weisman, Qian Xiao, Fan Zhang, Zhu Zhu, Andrew Cordle & Aaron Wyse

Supplementary Table 1. Demographics, disease activity status and histological features of lupus nephritis patients and healthy donors recruited as part of the AMP RA/SLE consortium.

	Healthy Donors (HD, n = 10)	SLE Patients (n = 21)
Age (years); median (IQR)	34 (23.25 – 38)	24 (18 – 38)
Sex (n, % female)	6 (60.0%)	17 (88.9%) (2 unspecified)
SLEDAI-2K*; median (IQR)	-	10 (8-16)
Current Severe Flare	-	10 (47.6%)
dsDNA Positive (n, %)	-	14 (66.7%)
Low Complement (n, %)	-	25 (59.5%)
Lupus Nephritis Class (n, %)	-	Class I/II: 1 (4,8%)<br>Class III/IV/mixed: 14 (66.6%)<br>Class V: 5 (23.8%)

Table of patient characteristics of the lupus nephritis patients included in the mass cytometry cohort. Values indicate medians with interquartile ranges (IQR) in parentheses or absolute numbers with relative values in parentheses. *SLEDAI-2K available in N = 10 patients. Abbreviations: SLEDAI-2K, Systemic Lupus Erythematosus Disease Activity Index 2000; IQR, Interquartile Range; dsDNA, anti-double stranded DNA antibodies.

Supplementary Table 2. Demographics, disease activity status and histological features of SLE patients and healthy donors with serum LAMP1 measurements.

	Healthy Donors (HD, n = 11)	SLE Patients (n = 67)
Age (years); median (IQR)	58.5 (27 – 70)	35 (25 – 50)
Sex (n, % female)	5 (45 %)	58 (86.6%)
SLEDAI-2K; median (IQR)	-	4 (2 –6)
dsDNA Level in U/ml *	-	59 (10 – 157)
dsDNA Positive (%) *	-	35 (66.0%)
Low Complement (%) **	-	25 (59.5%)
Lupus Nephritis Class (%)	-	No LN: 40 (59.7%)<br>History suggests LN, no biopsy: 5 (7.5%)<br>Class I/II: 7 (10.4%)<br>Class III/IV/mixed: 13 (19.4%)<br>Class V: 2 (2.9%)
Prednisolone Dose ***		0 mg/d: 20 (35.1%)<br>1-5 mg/d: 21 (36.8%)<br>>5 mg/d: 16 (28.07)
Additional Immunosuppressive Drugs (Combinations Possible) ***		Hydroxychloroquine: 25 (43.9%)<br>Mycophenolate Mofetil: 11 (19.3%)<br>Azathioprine: 9 (15.8%)<br>Methotrexate: 7 (12.3%)<br>Cyclosporin A: 2 (3.5%)<br>Rituximab: 2 (3.5%)<br>Cyclophosphamide: 1 (1.8%)<br>Leflunomide: 1 (1.8%)<br>Belimumab: 1 (1.8%)

Table of patient characteristics in whom soluble LAMP1 were measured in the serum. Values indicate medians with interquartile ranges in parentheses or absolute numbers with relative values in parentheses. *dsDNA levels available in N = 53 patients. **Complement levels available in N = 42 patients. ***Medication data available in N = 57 patients.

Supplementary Table 3. Patient characteristics for urinary proteomics

	Overall	HD	Class I/II	Proliferative	Mixed	Class V	VI
**n**	235	10	21	85	55	55	9
**Age (mean (SD))**	36.37 (11.76)	38.30 (14.82)	39.38 (11.93)	34.58 (11.30)	36.67 (11.72)	36.56 (11.53)	41.22 (13.78)
**Sex = F/M (%)**	202/33 (86.0/14.0)	8/2 (80.0/20.0)	20/1 (95.2/4.8)	76/9 (89.4/10.6)	44/11 (80.0/20.0)	46/9 (83.6/16.4)	8/1 (88.9/11.1)
**Race (%)**							
**Asian**	35 (14.9)	1 (10.0)	5 (23.8)	13 (15.3)	8 (14.5)	6 (10.9)	2 (22.2)
**Black**	99 (42.1)	3 (30.0)	11 (52.4)	32 (37.6)	18 (32.7)	30 (54.5)	5 (55.6)
**White**	72 (30.6)	6 (60.0)	4 (19.0)	28 (32.9)	20 (36.4)	13 (23.6)	1 (11.1)
**Other**	6 (2.6)	0 (0.0)	1 (4.8)	3 (3.5)	1 (1.8)	1 (1.8)	0 (0.0)
**Unknown**	23 (9.8)	0 (0.0)	0 (0.0)	9 (10.6)	8 (14.5)	5 (9.1)	1 (11.1)
**First Biopsy**	82 (34.9)	0 (0.0)	6 (28.6)	41 (48.2)	20 (36.4)	15 (27.3)	0 (0.0)
**Proliferative Subtype**							
**III**	78 (33.2)	0 (0.0)	0 (0.0)	46 (54.1)	32 (58.2)	0 (0.0)	0 (0.0)
**IV**	60 (25.5)	0 (0.0)	0 (0.0)	39 (45.9)	21 (38.2)	0 (0.0)	0 (0.0)
**NIH Activity Index* – median [IQR]**	4.00 [1.00, 8.00]		1.00 [0.00, 2.00]	5.00 [3.00, 9.00]	7.00 [5.00, 11.50]	0.00 [0.00, 1.00]	0.00 [0.00, 0.00]
**NIH Chronicity Index* – median [IQR]**	3.00 [1.00, 5.00]		3.00 [2.50, 4.00]	2.00 [1.00, 4.00]	3.00 [2.00, 5.25]	3.00 [1.00, 5.75]	9.00 [9.00, 9.00]
**Proteinuria (g/g Creatinine) – mean (SD)**	2.72 (2.39)		1.30 (1.09)	2.39 (1.74)	3.62 (3.23)	2.90 (2.42)	2.16 (1.13)
**Serum Creatinine – mean (SD)**	1.18 (0.82)	0.76 (0.15)	1.00 (0.43)	1.23 (0.91)	1.21 (0.77)	1.04 (0.61)	2.25 (1.37)
**Glomerular Filtration Rate in ml/min – mean (SD)**	85.94 (35.93)	109.07 (19.11)	88.64 (33.58)	84.03 (36.34)	83.26 (35.25)	94.23 (34.74)	38.43 (16.69)
**Low C3 Complement**	130 (60.2)		5 (31.2)	65 (79.3)	35 (64.8)	24 (43.6)	1 (11.1)
**Low C4 Complement**	107 (49.5)		7 (43.8)	55 (67.1)	28 (51.9)	17 (30.9)	0 (0.0)

Table of patient characteristics of the urine proteomic measurements. Values reported as n (%) unless otherwise indicated.

*: NIH Activity and Chronicity Index available for 154 patients.

Adapted from Fava *et al.* 2024 JCI Insight (doi:10.1172/jci.insight.172569) [30]

Supplementary Table 4. Mass cytometry panel

Antigen	Metal	Dilution	Antigen	Metal	Dilution
CD20	113In	1:100	CD11c	159Tb	1:100
CD3	115In	1:100	FPR1	160Gd	1:100
CD88	141Pr	1:100	IL13R	161Dy	1:100
CD11b	142Nd	1:100	CD56	162Dy	1:100
CD115	143Nd	1:100	CD85jILT2	163Dy	1:100
CD64	144Nd	1:100	CD10	164Dy	1:100
CD16	145Nd	1:100	CD177	165Ho	1:100
CD14	146Nd	1:100	CD107aLAMP1	166Er	1:100
CD13	147Sm	1:100	CD273PDL2	167Er	1:100
TLR4	148Nd	1:100	CD31	168Er	1:100
CD18a	149Sm	1:100	TLR6	169Tm	1:100
CD11ba	150Nd	1:100	CD54ICAM	170Er	1:100
CD279PD1	151Eu	1:100	CD66b	171Yb	1:100
CD170	152Sm	1:100	TIGIT	173Yb	1:100
CXCR1	153Eu	1:100	CD62L	174Yb	1:100
CD15	154Sm	1:100	CD172ab	175Lu	1:100
CD18	155Gd	1:100	CD302	176Yb	1:100
CD95Fas	156Gd	1:100	CD45	209Bi	1:100

Supplemental Figure 1. Compositional analysis of peripheral blood neutrophils in SLE and heathy donors. (**A**) Uniform Manifold Approximation and Projection (UMAP) plot demonstating the clustering and annotation of diverse immune cell types in the peripheral blood. (**B**) Dotplot of lineage markers of the immune cell type clusters (**C**) UMAP plots showing the signal intensity of 21 markers associated with neutrophil aging, maturation and activation status in peripheral blood neutrophils.

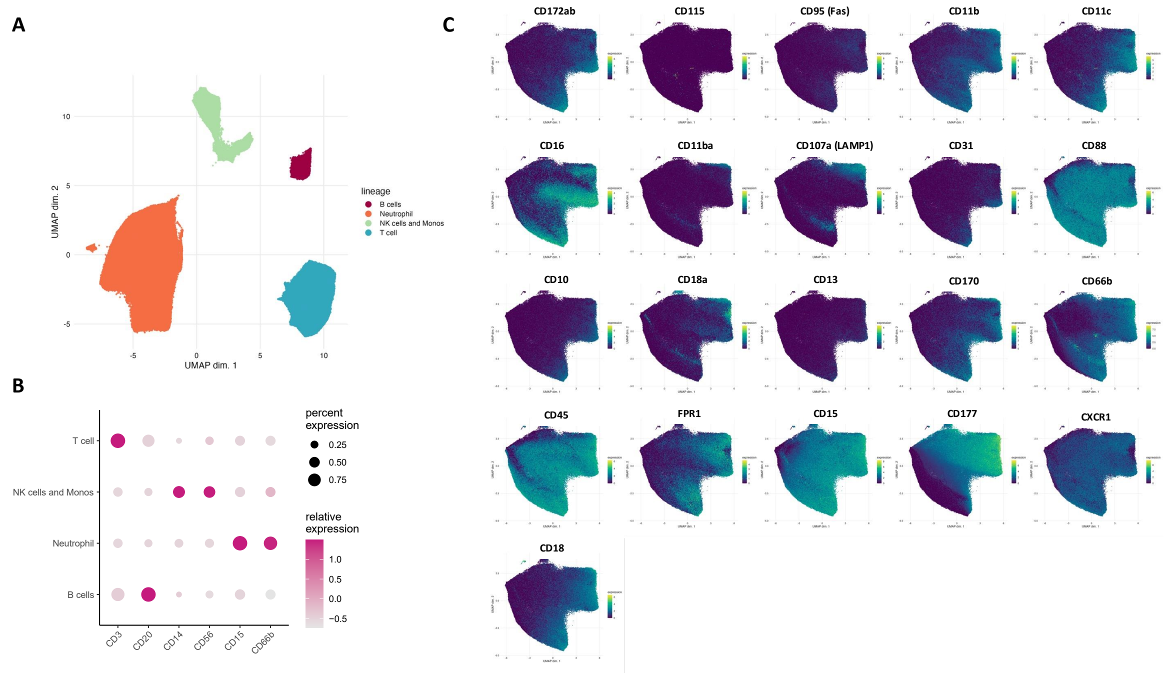

Supplemental Figure 2.

Analysis of *LAMP1* mRNA expression among 142 patients with SLE, comparing gene expression between SLE patients with and without lupus nephritis (LN) and between patients with active versus inactive LN.

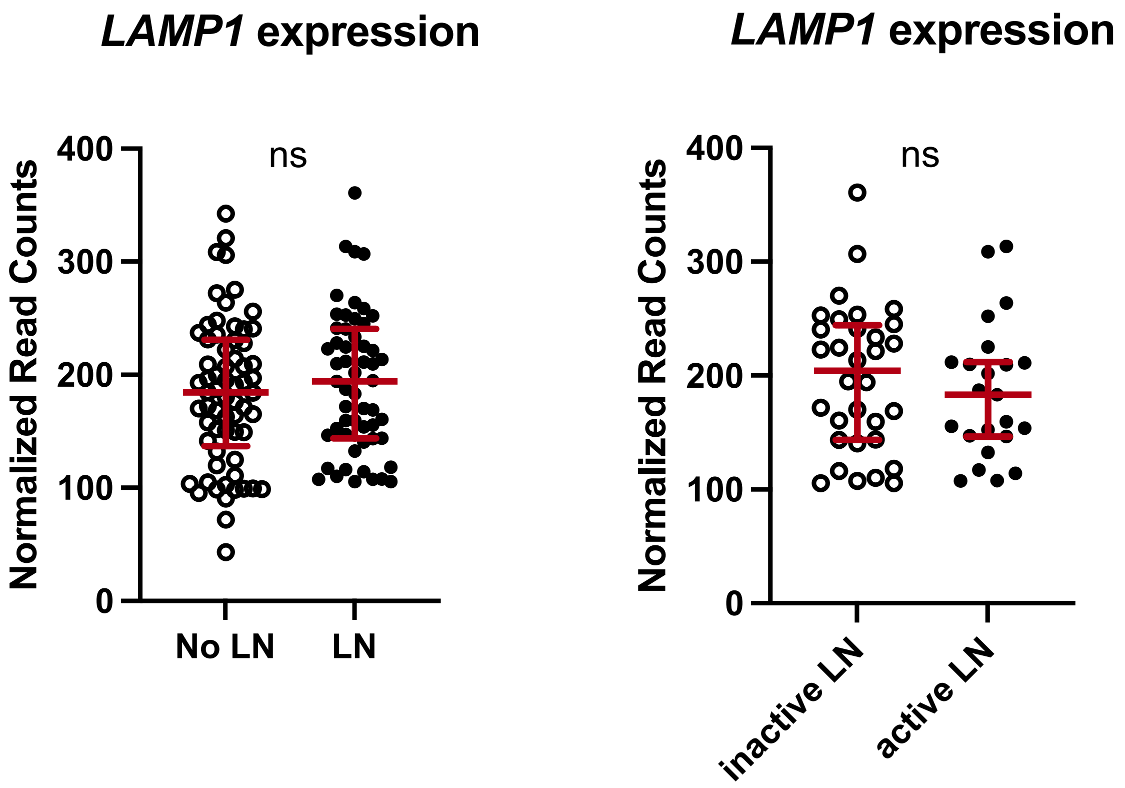

Supplemental Figure 3.

Confocal microscopy of healthy donor neutrophils stained with DAPI, CD15, LAMP1 and different granule markers, including MPO (primary/azurophilic granules), LTF (secondary/specific granules), CD35 (secretory granules) and colocalization of LAMP1 with the granule markers.

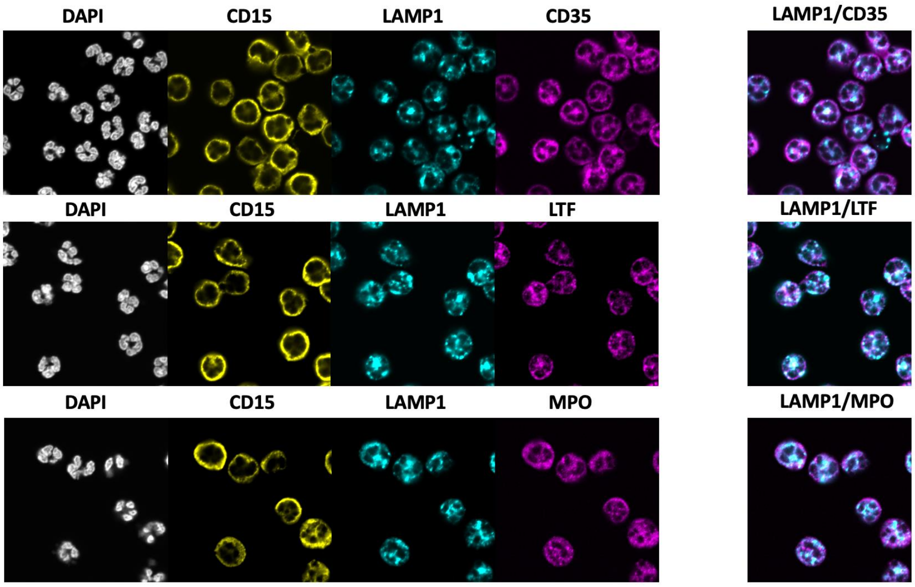

Supplemental Figure 4.

(A) Correlation of Serum soluble LAMP1 and donor age of both SLE patients and healthy donor, Pearson correlation. (B) Serum soluble LAMP1 levels across daily prednisolone equivalent doses.

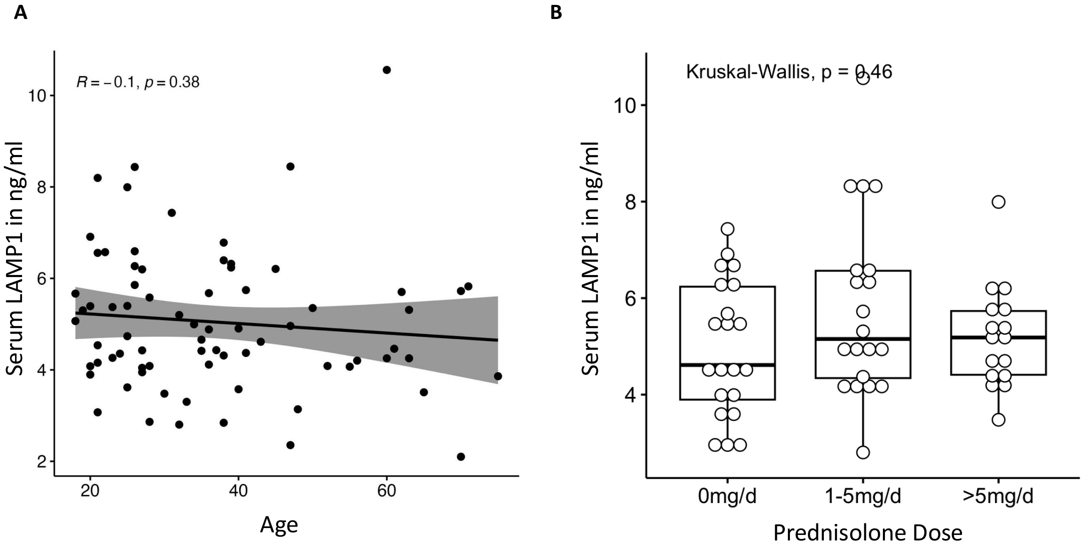

Supplement: Supplementary file 1 — Supporting File 1: eji70022‐sup‐0001‐SuppMat.pdf [file EJI-55-e70022-s001.pdf]
